# Mavacamten for Obstructive Hypertrophic Cardiomyopathy: Targeting Sarcomeric Hypercontractility with Demonstrated Long-Term Safety and Efficacy—A Narrative Review

**DOI:** 10.3390/jcm14238594

**Published:** 2025-12-04

**Authors:** Ghaith K. Mansour, Ali F. Altebainawi, Ahmad W. Hajjar, Sabry Babiker H. Sayed, Fares A. Alazem, Muhammad Raihan Sajid

**Affiliations:** 1 Department of Pharmaceutical Sciences, College of Pharmacy, Alfaisal University, Riyadh 11533, Saudi Arabia; gkmansour@alfaisal.edu; 2 Department of Clinical Pharmacy, College of Pharmacy, University of Hail, Hail 81422, Saudi Arabia; a.faris@uoh.edu.sa; 3 College of Medicine, Alfaisal University, Riyadh 11533, Saudi Arabia; ahmad_w_hajjar@hotmail.com (A.W.H.); falazem@alfaisal.edu (F.A.A.); 4 General Medicine Practice Program, Batterjee Medical College, Dammam 32313, Saudi Arabia; sabry.sayed@bmc.edu.sa

**Keywords:** hypertrophic cardiomyopathy, obstructive hypertrophic cardiomyopathy, mavacamten, cardiac myosin inhibitor, sarcomeric hypercontractility

## Abstract

Hypertrophic cardiomyopathy (HCM) is the most prevalent hereditary cardiovascular disorder characterized by unexplained left ventricular hypertrophy, sarcomeric hypercontractility, and dynamic left ventricular outflow tract (LVOT) obstruction in approximately 70% of patients. Current therapies predominantly offer symptomatic relief through indirect modulation of cardiac function, leaving the underlying molecular pathophysiology unaddressed. Mavacamten, a first-in-class, selective allosteric inhibitor of β-cardiac myosin ATPase, exemplifies a precision pharmacological approach by directly targeting the sarcomeric hypercontractility fundamental to obstructive HCM (oHCM). This review synthesizes extensive clinical and preclinical evidence delineating mavacamten’s mechanism of action, pharmacokinetics influenced by CYP2C19 genotype variability, and its demonstrated efficacy and long-term safety in improving functional capacity, symptom burden, and cardiac remodeling. Landmark trials, including EXPLORER-HCM and MAVERICK-HCM, underscore mavacamten’s ability to reduce LVOT gradients, enhance diastolic function, and lower cardiac biomarkers, heralding a paradigm shift from symptomatic management to disease-modifying therapy. Despite current knowledge gaps in long-term outcomes and diverse population responses, mavacamten establishes a critical foundation for molecularly targeted therapeutics in HCM and broader cardiomyopathies.

## 1. Introduction and Background

Hypertrophic cardiomyopathy (HCM) represents the most prevalent genetic cardiovascular disorder affecting humanity, with established epidemiological data demonstrating a global prevalence of approximately 1 in 500 individuals across all continents, ethnicities, and racial backgrounds [1]. This hereditary cardiac condition constitutes a complex genetic disorder characterized by unexplained left ventricular hypertrophy, microvascular ischemia, myocardial fibrosis, and significant diastolic dysfunction that fundamentally alters cardiac structure and function [2]. Contemporary epidemiological studies have revealed that the prevalence of HCM may be even higher when incorporating advanced genetic screening criteria, with some populations demonstrating rates approaching 0.2% of the general population, suggesting that millions of individuals worldwide are affected by this progressive cardiac disorder [3].

The pathophysiological foundation of HCM lies fundamentally within the cardiac sarcomere, the basic contractile unit of the myocardium, where over 1400 distinct genetic mutations have been identified across 11 or more genes encoding essential sarcomeric proteins [4]. The most commonly implicated genes include MYH7, encoding β-cardiac myosin heavy chain, and MYBPC3, encoding cardiac myosin-binding protein C, which together account for the majority of genetically identifiable cases of HCM [2]. These sarcomeric protein mutations fundamentally disrupt the normal regulation of actin–myosin cross-bridge formation and cycling, leading to excessive myosin–actin interactions that manifest as cardiac hypercontractility, the hallmark pathophysiological characteristic of HCM [5].

Obstructive hypertrophic cardiomyopathy (oHCM), present in approximately 70% of all HCM patients, represents the most clinically significant subset of the disease, characterized by dynamic left ventricular outflow tract (LVOT) obstruction that directly contributes to the symptom burden and functional limitations experienced by affected individuals [6]. The pathophysiology of LVOT obstruction involves complex hemodynamic alterations resulting from systolic anterior motion of the mitral valve, asymmetric septal hypertrophy, and abnormal papillary muscle morphology, creating a dynamic pressure gradient that varies with loading conditions and contractile state [7]. This obstruction not only impairs cardiac output but also elevates left ventricular filling pressures, contributing to the constellation of symptoms including dyspnea, chest pain, fatigue, syncope, and exercise intolerance that significantly impact patients’ quality of life and functional capacity [8].

The clinical presentation of oHCM encompasses a broad spectrum of symptoms and complications that range from asymptomatic disease discovered incidentally to severe heart failure symptoms and life-threatening arrhythmias [9]. Patients commonly experience exertional dyspnea, which may progress from mild exercise intolerance to severe functional limitation corresponding to New York Heart Association (NYHA) class III or IV symptoms [10]. Chest pain, often atypical and not necessarily related to coronary artery disease, frequently occurs due to myocardial ischemia resulting from increased oxygen demand, impaired coronary flow reserve, and microvascular dysfunction [11]. Syncope or presyncope, particularly with exertion, represents a concerning symptom that may herald increased risk of sudden cardiac death and often prompts urgent evaluation and intervention [4].

The natural history of HCM demonstrates significant heterogeneity, with some patients remaining asymptomatic throughout their lives while others develop progressive symptoms, heart failure, atrial fibrillation, and increased risk of sudden cardiac death [12]. Recent population-based studies from China have revealed an increasing incidence of HCM diagnosis, rising from 6.85 per 100,000 person-years in 2010 to 11.76 per 100,000 person-years in 2019, with projections suggesting a doubling of case numbers by 2029 if current trends continue [13]. This epidemiological trend may reflect improved diagnostic capabilities, increased awareness among healthcare providers, or genuine increases in disease prevalence, underscoring the growing clinical significance of HCM as a public health concern. Prior to the development of mavacamten, therapeutic options for oHCM remained severely limited and primarily focused on symptomatic management rather than targeting the underlying pathophysiological mechanisms [14]. Traditional pharmacological interventions included β-adrenergic receptor blockers and non-dihydropyridine calcium channel blockers, both aimed at reducing heart rate and myocardial contractility through extra-sarcomeric mechanisms [15]. While these agents could provide some symptomatic relief, their efficacy remained variable and unpredictable, with many patients continuing to experience significant functional limitations despite optimal medical therapy [16]. Disopyramide, a class IA antiarrhythmic agent with negative inotropic properties, represented another therapeutic option but was associated with significant side effects and poor tolerability, limiting its widespread clinical application [17].

For patients with severe symptoms refractory to medical therapy, invasive septal reduction therapies, including surgical septal myectomy and alcohol septal ablation, represented the primary therapeutic interventions capable of providing significant symptom improvement [18]. However, these procedures carry inherent risks associated with major cardiac interventions, require specialized expertise and centers, and are not suitable for all patients due to anatomical considerations or comorbidities [16]. Furthermore, these interventions address the consequences rather than the fundamental cause of LVOT obstruction, providing mechanical relief without modulating the underlying sarcomeric hypercontractility that drives the disease process.

The recognition of sarcomeric hypercontractility as the fundamental mechanism underlying HCM pathophysiology led to a revolutionary therapeutic approach targeting cardiac myosin directly [15]. This mechanistic understanding, coupled with advances in drug discovery and development, culminated in the creation of mavacamten (formerly MYK-461, now marketed as CAMZYOS), a first-in-class selective allosteric inhibitor of cardiac myosin ATPase [19]. Mavacamten represents a precision medicine approach to HCM treatment, designed specifically to target the motor protein responsible for excessive cardiac contractility and restore normal sarcomeric function [14].

The development of mavacamten marked a watershed moment in HCM therapeutics, representing the first pharmacological intervention capable of directly modulating sarcomeric function and addressing the root cause of disease pathophysiology [20]. On 28 April 2022, the United States Food and Drug Administration (FDA) granted approval for mavacamten for the treatment of adults with symptomatic NYHA class II-III obstructive hypertrophic cardiomyopathy to improve functional capacity and symptoms, marking the first disease-specific therapy approved for this condition [21]. This regulatory milestone represented the culmination of decades of basic science research, preclinical development, and rigorous clinical investigation that transformed our understanding of HCM from a purely descriptive diagnosis to a mechanistically targeted therapeutic intervention.

The approval of mavacamten has fundamentally altered the treatment paradigm for HCM, offering patients and clinicians a novel therapeutic option that directly targets the underlying molecular mechanisms responsible for disease pathophysiology [5]. Unlike traditional therapies that attempted to counteract the consequences of sarcomeric dysfunction through indirect mechanisms, mavacamten specifically inhibits the excessive myosin–actin cross-bridge formation that characterizes HCM, potentially offering more predictable and sustained therapeutic benefits [11]. The introduction of this targeted therapy has generated significant enthusiasm within the cardiovascular community and has opened new avenues for research into sarcomeric modulators and precision medicine approaches for inherited cardiac diseases.

**Figure 1:**
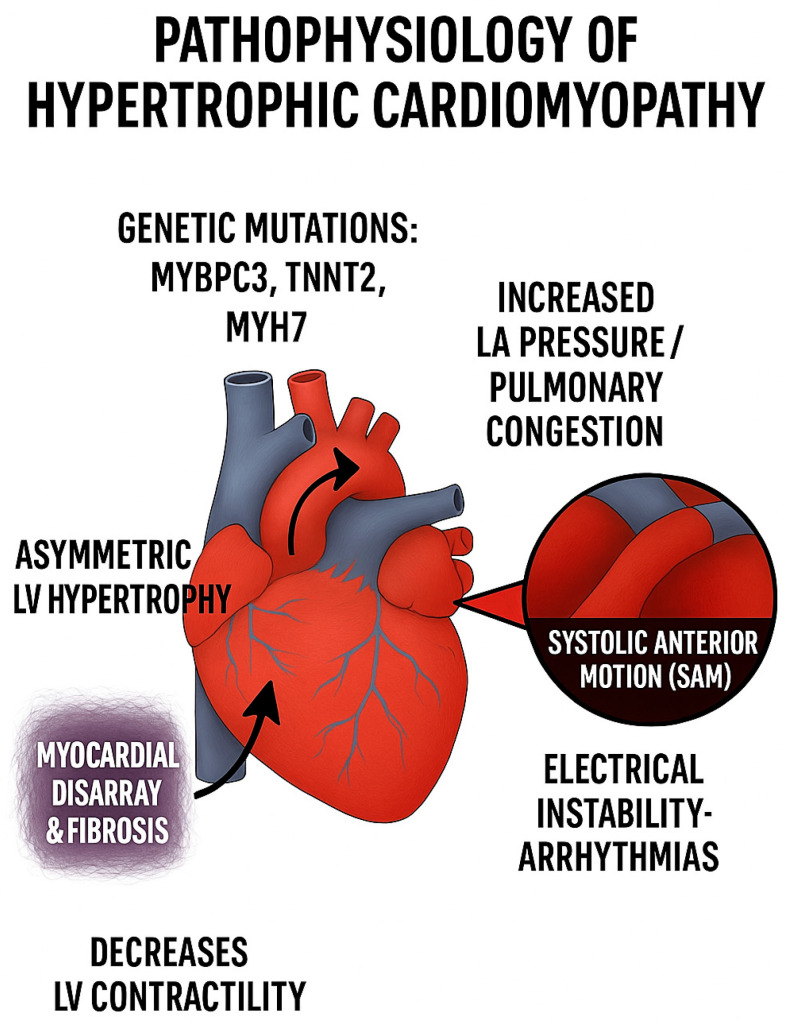
Pathophysiology of HCM.

The clinical significance of mavacamten extends beyond its immediate therapeutic effects, representing a proof-of-concept for targeting sarcomeric proteins in cardiovascular disease and paving the way for the development of additional cardiac myosin modulators [15]. The success of mavacamten has validated the therapeutic potential of directly modulating cardiac contractility at the molecular level and has stimulated renewed interest in sarcomere-targeted therapies for various cardiomyopathies and heart failure conditions. As our understanding of sarcomeric biology continues to evolve, mavacamten serves as a foundation for future drug development efforts aimed at addressing the root causes of cardiac dysfunction rather than merely managing symptoms. The pathophysiology of HCM with mechanism of action of mavacamten is shown in Figure 1.

## 2. Chemistry and Formulation on Mavacamten

Mavacamten exhibits a sophisticated molecular architecture specifically engineered to interact with the β-cardiac myosin motor domain through selective allosteric binding mechanisms. The compound possesses the chemical designation 3-(1-methylethyl)-6-[[(1S)-1-phenylethyl]amino]-2,4(1H,3H)-pyrimidinedione, with the molecular formula C_15_H_19_N_3_O_2_ and a molecular weight of 273.33 g/mol, classifying it within the pyrimidinedione pharmaceutical class. This precise molecular structure enables mavacamten to function as a reversible small-molecule allosteric inhibitor of cardiac myosin ATPase, distinguishing it from conventional cardiovascular therapeutics through its direct targeting of sarcomeric proteins rather than extra-sarcomeric pathways [22]. Mavacamten appears as a white to off-white powder and shows very low solubility in water and aqueous buffers across a pH range of 2 to 10. It is only sparingly soluble in alcohols such as methanol and ethanol but dissolves readily in organic solvents like DMSO and N-methyl-2-pyrrolidone (NMP) [23].

Mavacamten is provided in the form of immediate-release hard gelatin capsules. In addition to the active compound, the capsules include several inactive ingredients: croscarmellose sodium, hypromellose, magnesium stearate (non-bovine source), mannitol, and silicon dioxide. The capsule shell is composed of gelatin and is colored with black edible ink, black iron oxide, red iron oxide, titanium dioxide, and yellow iron oxide to provide its distinctive appearance [23].

**Figure 2:**
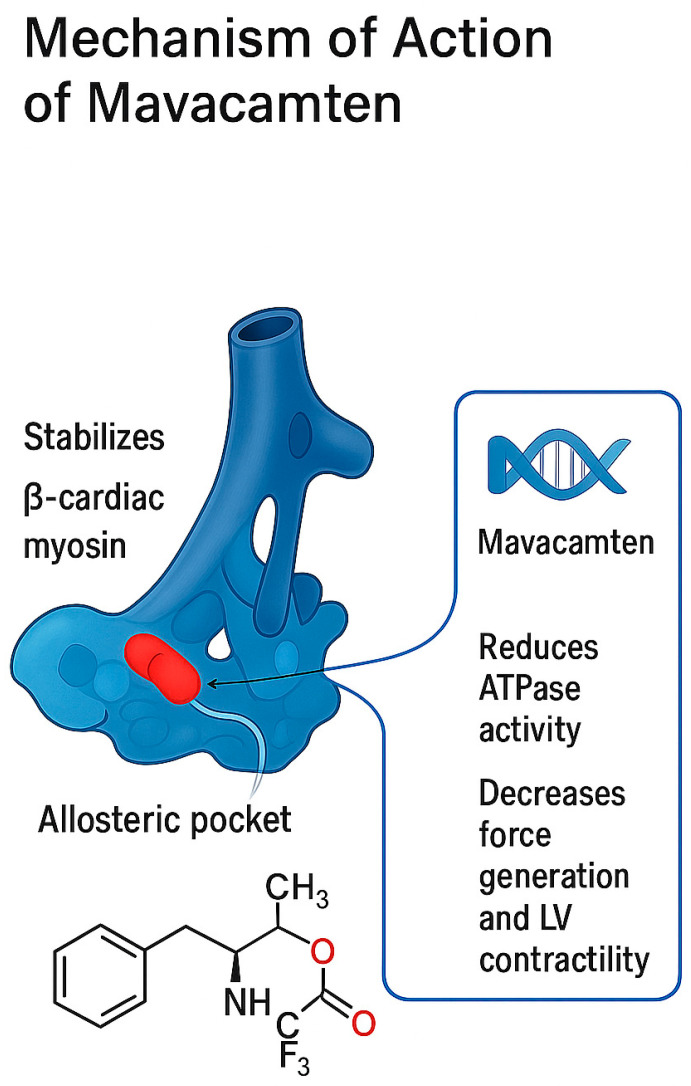
Mechanism of action of Mavacamten with its chemical structure.

Crystallographic studies have revealed that mavacamten binds to a specific allosteric pocket within the β-cardiac myosin motor domain, stabilizing a pre-stroke structural state that fundamentally alters the protein’s conformational dynamics. X-ray crystallography has demonstrated that mavacamten targets the same binding pocket as other myosin modulators, yet produces distinct allosteric effects through subtle differences in binding interactions and stabilized conformational states [24]. The compound’s binding induces conformational changes that propagate throughout the myosin molecule, affecting multiple aspects of the chemomechanical cycle and ultimately reducing the protein’s ATPase activity and force-generating capacity [19]. Figure 2 summarizes the mechanism of action of mavacamten.

The pharmaceutical formulation of mavacamten has been optimized to ensure reliable oral bioavailability and consistent therapeutic plasma concentrations. Mavacamten is available as hard capsules containing 2.5 mg, 5 mg, 10 mg, or 15 mg of mavacamten as the active pharmaceutical ingredient, providing flexible dosing options to accommodate individual patient needs and therapeutic responses [25]. The manufacturing process employs rigorous quality control measures to ensure batch-to-batch consistency and compliance with international pharmaceutical standards, reflecting the critical importance of precise dosing in this therapeutic indication.

## 3. Pharmacological Effects and Mechanism of Action

The pharmacological mechanism of mavacamten represents a revolutionary approach to cardiac therapeutics through direct modulation of sarcomeric contractility at the molecular level [19]. Mavacamten functions as a selective, reversible allosteric inhibitor of β-cardiac myosin ATPase, binding to a specific pocket within the myosin motor domain to reduce the rate of ATP hydrolysis and subsequent energy release available for actin–myosin cross-bridge formation. This mechanism directly addresses the fundamental pathophysiology of hypertrophic cardiomyopathy by normalizing the excessive sarcomeric activity that characterizes the disease, representing the first therapeutic intervention capable of targeting the root cause rather than merely managing symptoms [5].

Detailed kinetic analyses have revealed that mavacamten modulates multiple stages of the myosin chemomechanical cycle, extending beyond simple ATPase inhibition to affect various aspects of myosin function [19]. The compound significantly decreases the rate of phosphate release from the myosin–ADP-Pi complex, effectively trapping myosin in a weakly bound state that cannot generate force [19]. Additionally, mavacamten stabilizes an autoinhibited, super-relaxed state of two-headed cardiac myosin that is distinct from the single-headed motor fragment, creating a population of myosin molecules that are unavailable for productive interactions with actin [26]. This dual mechanism of action results in a dose-dependent reduction in both the number of force-generating cross-bridges and the kinetics of cross-bridge cycling, providing comprehensive modulation of cardiac contractility.

The super-relaxed state stabilized by mavacamten represents a physiologically relevant regulatory mechanism that is normally present in healthy cardiac muscle but becomes disrupted in HCM. In this state, myosin heads are folded back against the thick filament backbone in an energy-conserving configuration with extremely low ATPase activity, effectively sequestering them from actin interaction [27]. Mavacamten enhances the stability of this super-relaxed state, increasing the proportion of myosin molecules in this inactive configuration and thereby reducing the overall contractile capacity of the sarcomere [26]. This mechanism not only reduces contractility but also decreases myocardial energy consumption, potentially addressing the metabolic abnormalities associated with HCM. The selectivity of mavacamten for cardiac myosin represents a critical pharmacological advantage that distinguishes it from non-specific negative inotropic agents [20]. The compound demonstrates preferential binding to β-cardiac myosin heavy chain, the predominant isoform expressed in human ventricular myocardium, while showing minimal interaction with other myosin isoforms found in skeletal muscle or smooth muscle tissues [22]. This selectivity is achieved through specific molecular recognition of structural features unique to the cardiac myosin isoform, including distinct amino acid sequences and conformational characteristics within the binding pocket [24]. The result is a targeted therapeutic effect on cardiac contractility without significant interference with skeletal muscle function or other physiological processes dependent on different myosin isoforms.

The dose–response relationship for mavacamten demonstrates predictable and reversible effects on cardiac function, with contractile inhibition correlating directly with plasma drug concentrations [6]. At therapeutic concentrations, mavacamten produces clinically meaningful reductions in left ventricular outflow tract gradients while maintaining adequate systolic function for normal cardiac output [28]. The compound’s effects are fully reversible upon drug discontinuation, with contractile function returning to baseline levels as plasma concentrations decline, demonstrating the non-covalent and non-permanent nature of myosin inhibition [19]. This reversibility provides an important safety feature, allowing for dose adjustments or treatment discontinuation if adverse effects occur.

Preclinical studies in various animal models of hypertrophic cardiomyopathy have demonstrated that mavacamten not only reduces contractility but also promotes beneficial cardiac remodeling over time [11]. In mouse and pig models of HCM, chronic mavacamten treatment has been associated with reductions in left ventricular wall thickness, improvements in diastolic function, and decreased myocardial fibrosis [11]. These findings suggest that mavacamten may have disease-modifying effects beyond acute symptom relief, potentially altering the natural history of HCM through sustained modulation of sarcomeric activity and downstream signaling pathways [5].

The hemodynamic effects of mavacamten extend beyond simple contractile inhibition to include improvements in diastolic function and left ventricular relaxation [6]. By reducing the excessive contractile activation that characterizes HCM, mavacamten allows for more complete myocardial relaxation during diastole, potentially improving ventricular filling and reducing elevated filling pressures [29]. The compound’s effects on diastolic function may contribute significantly to symptom improvement, as diastolic dysfunction is a major contributor to exercise intolerance and heart failure symptoms in HCM patients [28].

Studies investigating the effects of mavacamten on myocardial energetics have revealed potential benefits in addressing the metabolic abnormalities associated with HCM. By promoting the super-relaxed state of myosin and reducing overall ATPase activity, mavacamten may improve the phosphocreatine-to-ATP ratio, a marker of myocardial energetic status [30]. These metabolic improvements may contribute to the drug’s therapeutic effects and could have long-term beneficial impacts on cardiac function and clinical outcomes.

## 4. Pharmacokinetics, Pharmacodynamics and Pharmacogenomics Behaviors of Mavacamten

The pharmacokinetic profile of mavacamten demonstrates characteristics optimized for once-daily oral dosing (Table S2) with predictable absorption, distribution, metabolism, and elimination patterns [31]. Following oral administration, mavacamten exhibits rapid absorption with peak plasma concentrations (Cmax) typically achieved within 0.6 to 1.5 h, indicating efficient gastrointestinal uptake and bioavailability [32]. The compound demonstrates moderate inter-individual variability in pharmacokinetic parameters, with this variability being significantly influenced by genetic polymorphisms in cytochrome P450 enzymes, particularly CYP2C19, which serves as the primary metabolic pathway for mavacamten clearance [32]. Therapy without CYP2C19 genotyping may cause imprecise dosing, which might result in decreased ejection fraction in poor metabolizers or a longer time to therapeutic dose in healthy individuals [33]. The initial dose for CYP2C19 poor metabolizers is 2.5 mg/day, with a daily maximum of 5 mg [34].

Hepatic cytochromes substantially metabolize mavacamten, and the CYP2C19 or CYP3A4 phenotype determines its half-life. Therefore, co-administration of drugs that either activate or inhibit these enzymes may drastically change the pharmacokinetics of mavacamten, perhaps resulting in reversible systolic dysfunction or subtherapeutic efficacy. Examples include: amlodipine, ranolazine, verapamil, and modafinil [35].

Population pharmacokinetic modeling incorporating data from 497 participants across 12 clinical studies has characterized the factors influencing mavacamten exposure and identified key covariates affecting drug disposition [31]. The apparent clearance (CL/F) of mavacamten is primarily determined by CYP2C19 phenotype, with poor metabolizers (1–57% prevalence) demonstrating significantly reduced clearance and correspondingly higher plasma concentrations compared to normal metabolizers (4–63%) [33]. Intermediate metabolizers (19–46% prevalence) exhibit pharmacokinetic parameters between those of poor and normal metabolizers, while ultra-rapid metabolizers (0–5%) may require higher doses to achieve therapeutic plasma concentrations [33].

The distribution characteristics of mavacamten indicate moderate tissue penetration with preferential accumulation in cardiac tissues [31]. The apparent volume of distribution suggests distribution beyond plasma volume, consistent with tissue binding and the compound’s lipophilic properties [32]. Protein binding studies have demonstrated high binding to plasma proteins, primarily albumin, which influences the compound’s distribution and elimination kinetics [31]. The steady-state pharmacokinetics of mavacamten are achieved within approximately one week of consistent dosing, allowing for predictable plasma concentrations during chronic therapy.

Metabolism of mavacamten occurs primarily through hepatic cytochrome P450 enzymes, with CYP2C19 serving as the predominant metabolic pathway [33]. CYP3A4 contributes to a lesser extent to mavacamten metabolism, particularly in individuals with reduced CYP2C19 activity [36]. The metabolic pathways involve oxidative processes that convert mavacamten to various metabolites, with the primary metabolites being pharmacologically inactive [31]. The elimination half-life of mavacamten varies significantly based on CYP2C19 phenotype, ranging from approximately 6–15 h in normal metabolizers to 23–37 h in poor metabolizers [32].

Mavacamten is metabolized primarily via oxidative processes by CYP2C19 into several metabolites. The three main metabolites identified have been demonstrated to be pharmacologically inactive in preclinical models [31]. Therefore, the therapeutic and adverse effects of mavacamten are attributed solely to the parent drug, and variability in metabolic activity influences exposure levels without generating active compounds that contribute to the response [31].

Pharmacogenomic considerations are particularly important for mavacamten therapy due to the substantial impact of CYP2C19 polymorphisms on drug exposure and clinical response [33]. The CYP2C19 gene exhibits significant genetic polymorphism across different ethnic populations, with varying frequencies of poor metabolizer alleles (*2, *3) and ultra-rapid metabolizer alleles (*17) [32]. Asian populations, including Chinese individuals, demonstrate higher frequencies of CYP2C19 poor metabolizer genotypes compared to Caucasian populations, necessitating ethnicity-specific dosing considerations [32]. The clinical significance of these pharmacogenomic differences has led to recommendations for CYP2C19 genotyping prior to mavacamten initiation to guide initial dosing and inform subsequent dose adjustments [31]. According to the American College of Cardiology/American Heart Association guidelines for PCI, routine use of CYP2C19 pharmacogenetic testing is not advised, but might be considered for patients showing high risk for poor clinical results. However, several US hospitals have advanced clinical CYP2C19 genotyping [37].

The pharmacodynamic effects of mavacamten demonstrate a clear concentration-response relationship, with increasing plasma concentrations correlating with greater reductions in left ventricular outflow tract gradients [31]. Population pharmacokinetic-pharmacodynamic modeling has established the relationship between mavacamten exposure and both efficacy endpoints (Valsalva LVOT gradient reduction) and safety parameters (left ventricular ejection fraction maintenance) [38]. These models support individualized dosing strategies based on patient-specific factors, including CYP2C19 genotype, baseline LVOT gradient, and left ventricular ejection fraction.

Drug–drug interaction studies have revealed that mavacamten has the potential to both affect and be affected by other medications through cytochrome P450 pathways [36]. Mavacamten exhibits weak to moderate induction of CYP3A4, as demonstrated by its effects on midazolam pharmacokinetics, with the magnitude of induction varying by CYP2C19 phenotype [36]. Strong CYP2C19 inhibitors, such as omeprazole and fluconazole, can significantly increase mavacamten exposure and may necessitate dose reductions to maintain safety [33]. Conversely, CYP2C19 inducers may reduce mavacamten concentrations and potentially compromise therapeutic efficacy.

The clinical implications of mavacamten’s pharmacokinetic variability have led to the development of sophisticated dosing algorithms that incorporate multiple patient factors [38]. Initial dosing recommendations are based on CYP2C19 phenotype, with poor metabolizers typically starting at lower doses (2.5 mg daily) compared to normal metabolizers (5 mg daily) [32]. Dose titration is guided by both pharmacokinetic considerations (plasma concentrations) and pharmacodynamic responses (echocardiographic parameters), with regular monitoring of left ventricular ejection fraction serving as a critical safety parameter [38].

Special population pharmacokinetics have been evaluated to ensure appropriate dosing across diverse patient groups [31]. Age-related changes in drug metabolism and clearance appear to have minimal impact on mavacamten pharmacokinetics within the typical HCM patient population, though elderly patients may require more frequent monitoring [32]. Gender differences in mavacamten pharmacokinetics are generally modest, though they may contribute to inter-individual variability in drug response [31]. Renal impairment has minimal impact on mavacamten clearance due to the drug’s primary hepatic metabolism, while hepatic impairment may significantly affect drug disposition and require dose adjustments.

The prolonged elimination half-life (23–37 h) and significantly reduced clearance in CYP2C19 poor metabolizers (PMs) lead to sustained higher plasma concentrations of mavacamten. This does not confer drug resistance; rather, it increases the risk of excessive pharmacologic effect. The primary concern is not reduced efficacy but a heightened and prolonged risk of adverse effects, particularly systolic dysfunction (reduced LVEF), due to persistent myosin inhibition. Consequently, the initial dosing for PMs is lower (2.5 mg/day), and they may require less frequent dose titration. The efficacy of mavacamten is not compromised by its longer stay in the bloodstream; in fact, PMs might achieve therapeutic targets at lower doses. However, the narrow therapeutic window necessitates careful and potentially more prolonged monitoring to ensure that efficacy (LVOT gradient reduction, symptom improvement) is achieved without crossing the threshold into toxicity. The need for re-titration is primarily a safety measure to avoid excessive drug accumulation, not a response to diminishing efficacy [31,39].

The development of therapeutic drug monitoring strategies for mavacamten reflects the importance of individualized dosing in achieving optimal clinical outcomes [38]. Plasma concentration targets have been established based on exposure-response relationships observed in clinical trials, with optimal efficacy typically achieved at steady-state concentrations of 350–700 ng/mL [31]. Regular pharmacokinetic monitoring, combined with echocardiographic assessment, enables clinicians to optimize dosing for individual patients while minimizing the risk of adverse effects related to excessive myosin inhibition.

## 5. Mavacamten in Clinical Trials

The clinical development program for mavacamten encompasses an extensive series of rigorous clinical trials (Table S1) designed to establish the drug’s safety and efficacy across multiple patient populations and clinical scenarios [21]. The systematic evaluation of mavacamten began with Phase 1 dose escalation studies in healthy volunteers to establish basic safety, tolerability, and pharmacokinetic parameters before progressing to patient populations with varying degrees of HCM severity and clinical manifestations [39]. Phase-1 studies in healthy volunteers are routine to establish pharmacokinetics, tolerability and safety margins prior to patient trials. These studies employed graded dose escalation with predefined stopping rules and intensive cardiac monitoring (serial ECG/echocardiography and PK sampling) to detect early systolic dysfunction. Importantly, mavacamten’s pharmacologic inhibition of myosin is reversible and contractile changes returned toward baseline after dose interruption/washout in preclinical and early human studies, mitigating but not eliminating the theoretical risk of excessive systolic depression in non-diseased myocardium. These safeguards justify the healthy-volunteer design while informing safe dosing for patient studies [39]. This comprehensive clinical investigation has generated robust evidence supporting mavacamten’s therapeutic utility and has formed the foundation for regulatory approvals worldwide.

The ODYSSEY-HCM study is the latest clinical trial that investigated mavacamten in symptomatic non-obstructive hypertrophic cardiomyopathy. An international phase 3 clinical trial, which is double-blind and placebo-controlled, aims to assess whether mavacamten enhances functional capacity and the health status reported by patients with symptomatic nonobstructive HCM [40]. 580 Patients were enrolled at 201 HCM referral centers across 22 countries between December 2022 and March 2024. The study focused on two main outcomes: the change in peak oxygen consumption from the start of the study to week 48, measured through cardiopulmonary exercise testing, and the change in the Kansas City Cardiomyopathy Questionnaire-Clinical Summary Score (KCCQ-CSS) over the same period. Notably, from the beginning of the study to week 48, the least-squares mean change in peak oxygen uptake was 0.52 mL per kilogram of body weight per minute (95% confidence interval [CI], 0.09 to 0.95) for the mavacamten group, compared to 0.05 mL per kilogram per minute (95% CI, −0.38 to 0.47) for the placebo group, resulting in a between-group difference of 0.47 mL per kilogram per minute (95% CI, −0.03 to 0.98; *p* = 0.07) [40]. The least-squares mean change in the KCCQ-CSS was 13.1 points (95% CI, 10.7 to 15.5) for those receiving mavacamten, while it was 10.4 points (95% CI, 8.0 to 12.8) for the placebo group, with a between-group difference of 2.7 points (95% CI, −0.1 to 5.6; *p* = 0.06) [40].

ODYSSEY-HCM (symptomatic non-obstructive HCM) did not demonstrate a statistically significant benefit in peak VO_2_ or KCCQ-CSS compared with placebo and had higher rates of treatment interruptions and some serious events in the mavacamten arm; thus, current evidence does not support robust symptomatic efficacy in non-obstructive HCM and further research is needed to define subgroups that might benefit [40].

In the safety data of the ODYSSEY-HCM, 42 patients (14.6%) in the mavacamten group and 15 patients (5.2%) in the placebo group experienced adverse events during treatment that led to a pause in the regimen. Additionally, 15 patients (5.2%) and 8 patients (2.8%) in the respective groups had adverse events that caused them to permanently stop the regimen. Serious adverse events, such as congestive heart failure and atrial tachyarrhythmias (fibrillation or flutter), were reported in 19 patients (6.6%) and 12 patients (4.2%) in the mavacamten group, and in 5 patients (1.7%) and 10 patients (3.4%) in the placebo group, respectively. In patients with nonobstructive HCM, mavacamten did not lead to a significantly greater enhancement in exercise capacity, as indicated by peak oxygen uptake, nor did it result in a notably greater reduction in symptoms according to the KCCQ-CSS, compared to a placebo [40].

The PIONEER-HCM study represented the first clinical investigation of mavacamten in patients with hypertrophic cardiomyopathy, serving as a proof-of-concept Phase 2 trial that demonstrated the drug’s potential therapeutic benefits. This open-label study enrolled 21 patients with obstructive HCM and evaluated the safety and efficacy of mavacamten over an extended treatment period, providing crucial preliminary data on the drug’s mechanism of action in human subjects [41]. The study demonstrated significant improvements in left ventricular outflow tract gradients, exercise capacity, and patient-reported outcomes, with most adverse effects being mild and irrelevant to mavacamten. Results showed no serious adverse effects related to mavacamten [41], establishing the foundation for subsequent larger randomized controlled trials and providing valuable insights into optimal dosing strategies and patient monitoring requirements.

The PIONEER-Open Label Extension (PIONEER-OLE) study provided long-term safety and efficacy data for 13 patients who completed the initial PIONEER-HCM trial, with some participants receiving mavacamten treatment for over 200 weeks. This extended follow-up demonstrated sustained therapeutic benefits with continued improvements in KCCQ and maintenance of reduced LVOT gradients over years of treatment. The long-term safety profile remained favorable, with the majority of adverse events being mild (65.3%) to moderate (27.8%) in severity like: fatigue, arthralgia, nasopharyngitis, upper respiratory tract infection, and only one patient experiencing an isolated reduction in left ventricular ejection fraction below 50%, which resolved with dose adjustment [41].

The EXPLORER-HCM trial stands as the pivotal Phase 3 randomized, double-blind, placebo-controlled study that served as the primary basis for mavacamten’s regulatory approval [42]. This landmark study enrolled 251 patients with symptomatic obstructive HCM across multiple international centers, randomizing them to receive either mavacamten or placebo for 30 weeks [43]. The primary composite endpoint combined objective measures of functional capacity (peak oxygen consumption) and subjective symptom assessment (NYHA functional class), providing a comprehensive evaluation of therapeutic benefit [42]. The study met its primary endpoint, with 45 patients (37%) in the mavacamten group achieving the composite endpoint compared to 22 patients (17%) in the placebo group, representing a statistically significant and clinically meaningful difference.

Secondary endpoints in EXPLORER-HCM demonstrated the breadth of mavacamten’s therapeutic effects, with significant improvements observed across multiple domains of HCM pathophysiology and patient experience [43]. Patients receiving mavacamten showed substantial reductions in post-exercise LVOT gradients, improvements in KCCQ-CSS, and reductions in cardiac biomarkers including NT-proBNP and high-sensitivity cardiac troponin I [44]. The magnitude of these improvements exceeded the minimal clinically important differences established for these endpoints, indicating that the statistical significance translated into meaningful clinical benefits for patients. Patients receiving mavacamten and those receiving a placebo had similar overall incidence of adverse events, major adverse events, and cardiac adverse events, including AF. The agent’s safety and tolerability were comparable to those of a placebo. In the placebo group, one patient died unexpectedly [43].

The MAVA-LTE (Long-Term Extension) study provided crucial long-term safety and efficacy data by following 231 patients from both EXPLORER-HCM and MAVERICK-HCM who continued mavacamten therapy for up to 180 weeks. This extension study demonstrated sustained therapeutic benefits with continued improvements in symptoms, functional capacity, and hemodynamic parameters over nearly three and a half years of treatment. The long-term safety profile remained consistent with shorter-term studies, with less than 10% of patients experiencing transient reductions in left ventricular ejection fraction below 50%, 7.8% de novo Atrial fibrillation, and only 1.3% requiring permanent treatment discontinuation [45].

The VALOR-HCM trial addressed a critical clinical question by evaluating mavacamten’s ability to reduce the need for septal reduction therapy in patients with severely symptomatic obstructive HCM who met guideline criteria for invasive intervention [18]. This randomized, double-blind, placebo-controlled study enrolled 112 patients who were deemed eligible for septal reduction therapy based on their symptom severity and hemodynamic parameters [18]. The study design included a unique crossover component where patients initially randomized to placebo crossed over to active treatment at 16 weeks, allowing for comprehensive evaluation of mavacamten’s effects in this high-risk population.

VALOR-HCM results demonstrated mavacamten’s remarkable ability to obviate the need for septal reduction therapy, with the majority of patients no longer meeting guideline criteria for invasive intervention following treatment initiation [18]. At 56 weeks, only 10 of 108 evaluable patients (9.3%) had undergone septal reduction therapy, with 5 in the original mavacamten group and 5 in the placebo crossover group [18]. The sustained nature of this benefit was confirmed by extended follow-up at 128 weeks, where nearly 90% of patients remained on long-term mavacamten therapy without requiring invasive procedures [46]. The most common relevant adverse events were COVID-19 (37.9%), fatigue (16.7%), and dizziness (14.8%), while 19.4% of patients experienced serious treatment-emergent adverse events, and 2.8% permanently discontinued the drug due to adverse events that met protocol criteria [46].

The MAVERICK-HCM study specifically evaluated mavacamten’s effects in 59 patients with non-obstructive HCM, addressing an important subset of the HCM population that lacks significant LVOT obstruction but may still experience symptoms and functional limitations [47]. While the study did not demonstrate significant improvements in the primary endpoint of peak oxygen consumption, it did show meaningful reductions in NT-proBNP and high-sensitivity cardiac troponin I, suggesting potential benefits in this patient population that warrant further investigation. There were no differences in reported serious adverse events between treatment groups (10% on mavacamten and 21% on placebo), indicating that the medication was well tolerated. Atrial fibrillation was a common serious adverse event (5% in both groups), while the majority of other side effects (>70%) were minor [47].

The EXPLORER-CN trial represents an important regional study designed to evaluate mavacamten’s efficacy and safety specifically in Chinese patients with symptomatic obstructive HCM [48]. This Phase 3, randomized, double-blind, placebo-controlled study enrolled approximately 81 participants across multiple centers in China, addressing potential ethnic and genetic differences in drug response and safety profile [48]. The study results demonstrated efficacy and safety consistent with the global EXPLORER-HCM population, with significant improvements in LVOT gradients and echocardiographic parameters observed in Chinese patients receiving mavacamten [49]. The mavacamten and placebo groups saw comparable rates of treatment-emergent adverse events. Two patients in the placebo group stopped therapy early, one for personal reasons and the other because of COVID-19-related problems [49].

Subgroup analyses across the major clinical trials have provided valuable insights into mavacamten’s effects in specific patient populations and clinical scenarios [50]. Gender-specific analyses from EXPLORER-HCM revealed that while women entered the study with more severe symptoms and functional limitations compared to men, they experienced similar therapeutic benefits from mavacamten treatment [50]. Interestingly, women demonstrated greater improvements in certain patient-reported outcomes, including KCCQ-CSS and NT-proBNP reductions, suggesting potential gender-related differences in treatment response that may reflect underlying disease characteristics.

Age-related subgroup analyses have examined mavacamten’s effects across different age groups, providing important data for clinical decision-making in both younger and older patients [51]. While the majority of clinical trial participants were middle-aged adults, analyses of younger and older patient subgroups have suggested consistent therapeutic benefits across age ranges, though individual dose adjustments may be necessary based on age-related changes in drug metabolism and cardiac function [51]. These findings support mavacamten’s use across the typical age spectrum of HCM patients while emphasizing the importance of individualized treatment approaches.

The integration of echocardiographic outcomes across clinical trials has provided comprehensive data on mavacamten’s structural and functional cardiac effects [52]. Beyond the primary hemodynamic endpoints of LVOT gradient reduction, studies have demonstrated improvements in diastolic function parameters, reductions in left atrial volume index, and evidence of reverse cardiac remodeling over time [49]. These structural improvements suggest that mavacamten may have disease-modifying effects beyond acute symptom relief, potentially altering the natural history of HCM through sustained modulation of cardiac structure and function.

Safety analyses across the clinical trial program have established mavacamten’s overall favorable risk-benefit profile while identifying key monitoring requirements for clinical practice. Integrated safety data from over 368 patients across five clinical studies, with cumulative exposure exceeding 527 patient-years, have demonstrated that treatment-emergent adverse events are generally mild to moderate in severity. The most significant safety consideration involves monitoring for reductions in left ventricular ejection fraction, which occurred in approximately 10% of patients but was typically reversible with dose adjustment or temporary treatment interruption [53].

Pediatric clinical development of mavacamten is currently underway with the SCOUT-HCM trial, a Phase 3 randomized, double-blind, placebo-controlled study designed to evaluate the drug’s efficacy, safety, and pharmacokinetics in adolescents aged 12 to less than 18 years with symptomatic obstructive HCM. This study will enroll 40 adolescent patients and represents the first prospective evaluation of cardiac myosin inhibition in the pediatric HCM population [54]. The primary endpoint focuses on changes in Valsalva LVOT gradient, with secondary endpoints including other echocardiographic parameters, safety assessments, pharmacokinetic characterization, and exercise capacity measurements tailored to the adolescent population.

Real-world evidence studies have begun to complement the clinical trial data by evaluating mavacamten’s performance in routine clinical practice settings. These studies have generally confirmed the efficacy and safety findings observed in clinical trials while providing insights into the practical aspects of mavacamten use, including patient selection, dose titration strategies, and long-term monitoring approaches [55]. Real-world data have also revealed the drug’s impact on healthcare utilization, with reductions in hospitalizations and emergency department visits observed in some patient cohorts receiving mavacamten therapy.

While the clinical trial program for mavacamten is robust, it is important to acknowledge its limitations regarding external validity. The pivotal EXPLORER-HCM trial primarily enrolled patients with symptomatic obstructive HCM (NYHA class II-III), excluding those with advanced heart failure (NYHA IV) or severe comorbidities. Furthermore, over 90% of participants in EXPLORER-HCM were White, raising questions about the generalizability of findings to more ethnically diverse populations. The MAVERICK-HCM and ODYSSEY-HCM trials investigated non-obstructive HCM but yielded equivocal or negative results for primary efficacy endpoints, leaving the therapeutic role in this subset uncertain. These selective enrollment criteria mean that the efficacy and safety profile of mavacamten in underrepresented ethnic groups, patients with end-stage disease, and the broader non-obstructive HCM population are not fully characterized. Post-marketing registries and real-world evidence studies are crucial to confirm the drug’s performance in these diverse clinical scenarios and to guide broader clinical adoption.

## 6. Cost-Effectiveness and Pharmacoeconomics

The Institute for Clinical and Economic Review (ICER) conducted the most comprehensive cost-effectiveness analysis of mavacamten using a semi-Markov decision analytic model with a lifetime time horizon [56]. Using a placeholder price of $75,000 per year, the model demonstrated that mavacamten produces more quality-adjusted life-years (QALYs) than standard first-line therapy but at substantially higher costs [56]. The incremental cost-effectiveness ratio (ICER) for mavacamten compared to standard treatment was approximately $1,200,000 per QALY gained, far exceeding conventional cost-effectiveness thresholds of $50,000–$100,000 per QALY [56]. When compared to disopyramide, the ICER increased to $1,500,000 per QALY, indicating even less favorable cost-effectiveness [56]. 

The economic evaluation reveals that septal reduction procedures, including surgical myectomy and alcohol septal ablation, demonstrate superior cost-effectiveness compared to mavacamten [56]. In the ICER analysis, both septal ablation and myectomy “dominated” mavacamten, meaning they provided better outcomes at lower costs [56]. Recent real-world data comparing hospital costs for septal reduction therapies shows that alcohol septal ablation procedures result in shorter length of stay (6.6 vs. 10.9 days) and lower total hospitalization costs ($240,792 vs. $314,359) compared to surgical myectomy with implantable cardioverter defibrillator placement. These procedural costs are significantly lower than the annual treatment costs associated with mavacamten therapy [57].

The economic model based treatment effects on changes in New York Heart Association (NYHA) functional class, which was considered the most valid approach for translating study results across treatment options into quality of life improvements [56]. The model findings were identical whether health gains were summarized with QALYs or equal value life-years gained (evLYG), as mortality effects were primarily associated with perioperative risks from septal reduction procedures [56]. 

The cost-effectiveness analysis faced several significant limitations. The model was based on only 30 weeks of data from the EXPLORER trial, creating substantial uncertainty about long-term treatment effects and safety outcomes [56]. The evidence for comparator treatments (myectomy, septal ablation, and disopyramide) came from observational studies rather than randomized controlled trials, potentially affecting the validity of indirect comparisons [56]. Additionally, more than 90% of patients in the EXPLORER trial were White, raising questions about generalizability to diverse patient populations and potential differential treatment effects [56].

The high annual cost of mavacamten therapy presents significant budget impact concerns for healthcare systems. At the placeholder price of $75,000 annually, widespread adoption could substantially increase healthcare expenditures for HOCM management [56]. The economic burden is particularly concerning given the chronic nature of HOCM requiring long-term treatment. The ICER analysis emphasized that mavacamten pricing should align with transparent estimates of treatment benefits for patients and families, with pricing moderated to reflect uncertainty about longer-term safety until additional outcomes data become available [56]. This recommendation underscores the need for value-based pricing strategies that balance clinical innovation with economic sustainability.

Real-world studies demonstrate that mavacamten provides comparable hemodynamic and functional improvements to septal reduction therapies, with both achieving greater than 70% reduction in Valsalva left ventricular outflow tract gradients. However, the economic implications of these equivalent clinical outcomes favor the one-time procedural interventions over chronic medical therapy from a cost-effectiveness perspective [58]. The economic evaluation of mavacamten would benefit from longer-term clinical data, direct comparative effectiveness studies with septal reduction procedures, and real-world evidence on healthcare resource utilization patterns [56]. Additionally, research on optimal patient selection criteria could improve cost-effectiveness by identifying subgroups most likely to benefit from mavacamten therapy. The establishment of post-approval clinical registries to assess efficacy in more diverse populations and detect rare side effects represents an important component of ongoing pharmacoeconomic evaluation [56]. 

Current evidence suggests that mavacamten, while clinically effective for obstructive hypertrophic cardiomyopathy, faces significant cost-effectiveness challenges at current pricing levels. The ICERs substantially exceed standard thresholds, and septal reduction procedures demonstrate superior economic value. Future pricing strategies and additional clinical evidence will be critical for establishing mavacamten’s role in value-based HOCM management.

## 7. Toxicological Profile of Mavacamten

The comprehensive toxicological evaluation of mavacamten has encompassed extensive preclinical studies designed to characterize the drug’s safety profile across multiple organ systems and identify potential risks associated with chronic cardiac myosin inhibition [53]. Preclinical toxicology studies in various animal species, including rodents and non-human primates, have evaluated the compound’s effects on cardiac function, skeletal muscle performance, and other physiological systems at doses substantially exceeding those used clinically [39]. These studies have generally demonstrated an acceptable safety margin for mavacamten, with the primary toxicological concerns relating to excessive cardiac myosin inhibition leading to reduced cardiac contractility rather than direct organ toxicity [20].

Cardiac toxicology represents the most significant safety consideration for mavacamten, given its mechanism of action involves direct inhibition of the heart’s primary contractile protein [53]. Preclinical studies have established dose–response relationships for cardiac effects, identifying therapeutic windows where clinically beneficial reductions in hypercontractility can be achieved without compromising essential cardiac function [59]. At higher doses, mavacamten can produce excessive myosin inhibition leading to systolic dysfunction and reduced cardiac output, emphasizing the importance of careful dose titration and patient monitoring in clinical practice [6].

Genotoxicity studies for mavacamten consistently demonstrated negative results, indicating that mavacamten does not possess significant genotoxic potential under the conditions tested [20]. The absence of genotoxicity reduces concerns about long-term carcinogenic risk, though longer-term clinical surveillance remains important given the relatively recent introduction of the drug to clinical practice.

Reproductive toxicology studies have evaluated mavacamten’s effects on fertility, embryonic development, and fetal outcomes in pregnant animals. These studies have identified potential concerns regarding fetal development, leading to recommendations against mavacamten use during pregnancy unless the maternal benefit clearly outweighs potential fetal risks [20]. The mechanism underlying these developmental effects may relate to the importance of myosin function in cardiovascular development, highlighting the need for effective contraception in women of childbearing potential receiving mavacamten therapy.

Skeletal muscle toxicity represents a theoretical concern given mavacamten’s mechanism of action, though preclinical studies have demonstrated selectivity for cardiac versus skeletal muscle myosin isoforms. In vitro studies using rabbit psoas muscle preparations have shown that mavacamten has differential effects on cardiac versus skeletal muscle contractility, with much higher concentrations required to affect skeletal muscle function [59]. Clinical monitoring has not revealed significant skeletal muscle toxicity in patients receiving therapeutic doses of mavacamten, supporting the compound’s selectivity for cardiac myosin under clinical conditions.

The evaluation of mavacamten’s effects on other organ systems has encompassed comprehensive assessments of renal, respiratory, gastrointestinal, and neurological function [53]. Preclinical studies have not identified significant toxicological concerns in these organ systems, and clinical trial experience has not revealed concerning safety signals beyond the expected pharmacological effects on cardiac contractility [39]. This broad safety profile supports mavacamten’s use in patients with multiple comorbidities, though individual patient assessment remains important for optimizing therapeutic outcomes.

The development of a comprehensive risk evaluation and mitigation strategy (REMS) for mavacamten reflects the importance of managing the drug’s most significant safety risks through structured monitoring and education programs. The REMS program focuses primarily on monitoring for reductions in left ventricular ejection fraction and ensuring appropriate patient selection and follow-up [60]. Healthcare providers must complete educational training and patients must undergo regular echocardiographic monitoring to participate in the REMS program 4, 8, and 12 weeks following the start of treatment, and then every 12 weeks after that, creating a framework for safe and effective mavacamten use in clinical practice [60].

## 8. Safety Profile, Adverse Events, and Long-Term Outcomes

The safety profile of mavacamten has been extensively characterized through integrated analyses of multiple clinical trials encompassing over 368 patients with cumulative exposure exceeding 527 patient-years, providing robust data on both short-term and long-term safety considerations. The most comprehensive safety analysis includes patients from PIONEER-HCM, EXPLORER-HCM, VALOR-HCM, and their respective long-term extension studies, with follow-up periods extending up to 180 weeks in some participants [53]. This extensive safety database demonstrates that mavacamten is generally well-tolerated, with the majority of adverse events being mild to moderate in severity and consistent with the drug’s known pharmacological effects [61].

The most clinically significant safety concern associated with mavacamten therapy involves reductions in left ventricular ejection fraction (LVEF), which represents the expected pharmacological consequence of cardiac myosin inhibition when dosing exceeds optimal therapeutic levels [53]. Integrated safety analyses have revealed that approximately 10% of patients experience transient reductions in LVEF below 50% during treatment, with these events typically being reversible through dose reduction or temporary treatment interruption. Importantly, the majority of patients experiencing LVEF reductions can continue mavacamten therapy at adjusted doses, and only 1.3% of patients require permanent treatment discontinuation due to persistent systolic dysfunction [45].

Systematic meta-analyses of mavacamten safety data across randomized controlled trials have demonstrated that treatment-emergent adverse events occur at similar frequencies in mavacamten and placebo groups, with no significant increase in serious adverse events attributable to the drug [62]. A comprehensive meta-analysis of six randomized controlled trials involving 732 patients found no significant difference between mavacamten and placebo groups in the incidence of total treatment-emergent adverse events or serious adverse events, supporting the drug’s overall favorable safety profile [63]. However, mavacamten treatment was associated with a statistically significant increase in the risk of LVEF reduction below 50%, confirming this as the primary safety concern requiring clinical monitoring.

The long-term safety profile of mavacamten has been particularly well-characterized through the PIONEER-OLE study, which has followed patients for over 200 weeks of continuous treatment. This extended follow-up has demonstrated that adverse events remain predominantly mild to moderate in severity over time, with no evidence of cumulative toxicity or emerging safety signals with prolonged exposure [41]. Only one patient in this long-term study experienced an isolated reduction in LVEF below 50%, which resolved with dose adjustment, supporting the safety of extended mavacamten therapy when appropriate monitoring is maintained.

Real-world safety data from the mavacamten Risk Evaluation and Mitigation Strategy (REMS) database, covering 22 months of post-marketing experience, have provided important insights into safety outcomes in routine clinical practice. Analysis of data from April 2022 to February 2024 revealed that the need for temporary treatment interruption due to LVEF reduction below 50% was low, including among patients receiving therapy for more than one year [60]. Even fewer patients experienced LVEF reductions associated with heart failure symptoms requiring hospitalization, confirming that the risk of clinically significant systolic dysfunction is manageable with appropriate monitoring protocols.

Cardiovascular adverse events beyond LVEF reduction have been systematically evaluated across the clinical trial program, with particular attention to arrhythmias, heart failure, and sudden cardiac death. Exposure-adjusted incidence rates for adjudicated cardiovascular events in EXPLORER-HCM and MAVA-LTE studies have shown rates of heart failure (1.42 per 100 patient-years) and new-onset atrial fibrillation (1.89 per 100 patient-years) that are consistent with the natural history of HCM rather than representing drug-related toxicity [64]. No increase in sudden cardiac death or ventricular arrhythmias has been observed with mavacamten treatment, supporting the drug’s cardiovascular safety profile.

Electrophysiological safety assessments have revealed interesting patterns of arrhythmia occurrence during mavacamten therapy, with some studies suggesting transient increases in certain arrhythmia types early in treatment followed by stabilization over time. Holter monitoring studies in HCM patients treated with mavacamten have shown transient arrhythmic fluctuations during the first six months of treatment, followed by long-term electrophysiological stabilization [65]. These findings support the need for early rhythm monitoring but also suggest that mavacamten’s long-term electrophysiological effects are generally benign and may even be beneficial through reduction in the arrhythmogenic substrate associated with HCM.

Gastrointestinal adverse events represent a notable category of side effects observed with mavacamten therapy, though they are generally mild and do not require treatment discontinuation [53]. The most commonly reported gastrointestinal adverse events include nausea, diarrhea, and abdominal discomfort, which may be related to the drug’s formulation or indirect effects on cardiovascular hemodynamics [62]. These events typically resolve with continued treatment or can be managed through dose adjustments or supportive care measures without compromising therapeutic efficacy.

Neurological adverse events, particularly dizziness and headache, have been reported in clinical trials and may be related to hemodynamic changes associated with improved cardiac function and reduced LVOT obstruction [53]. The incidence of dizziness was higher in mavacamten-treated patients compared to placebo in some studies, possibly reflecting the hemodynamic adjustments that occur as LVOT gradients are reduced and cardiac output patterns change [66]. These events are typically transient and may actually represent a positive indicator of therapeutic response rather than true drug toxicity.

Drug discontinuation rates due to adverse events have remained low across clinical trials, with only 10.9% of patients permanently discontinuing treatment in integrated safety analyses. Among those who discontinued treatment, only 13 patients (3.5% of the total treated population) did so specifically due to adverse events, with the remainder discontinuing for other reasons including protocol completion, patient preference, or non-safety related factors [53]. This low discontinuation rate supports mavacamten’s overall tolerability and suggests that most patients can continue long-term therapy when appropriately monitored.

The relationship between mavacamten dose and adverse event incidence has been systematically evaluated to inform optimal dosing strategies and risk management approaches. Population pharmacokinetic-pharmacodynamic modeling has established exposure-response relationships for both efficacy and safety endpoints, demonstrating that optimal therapeutic outcomes can be achieved within a well-defined exposure range that maximizes efficacy while minimizing the risk of LVEF reduction [38]. These findings support individualized dosing approaches based on patient-specific factors including CYP2C19 genotype, baseline cardiac function, and hemodynamic response to treatment.

Special population safety considerations have been evaluated for patients with various comorbidities and demographic characteristics [53]. Elderly patients may require more frequent monitoring due to age-related changes in drug metabolism and increased susceptibility to hemodynamic changes, while patients with atrial fibrillation or other arrhythmias may need specialized cardiac rhythm monitoring during treatment initiation [66]. Gender-specific safety analyses have not revealed significant differences in adverse event patterns between men and women, though individual patient factors may influence treatment tolerance and monitoring requirements.

Long-term cardiovascular outcomes data are beginning to emerge from extended follow-up studies, providing insights into mavacamten’s effects on disease progression and major cardiovascular events [46]. While the follow-up period remains relatively short compared to the natural history of HCM, preliminary data suggest that mavacamten may have beneficial effects on cardiovascular outcomes beyond symptom improvement, including potential reductions in heart failure hospitalizations and maintenance of stable cardiac function over time [41]. However, longer-term studies will be necessary to definitively establish mavacamten’s impact on major cardiovascular endpoints and overall survival in HCM patients. Based on the provided trial protocols, mavacamten contraindications included a recent history of syncope or sustained ventricular tachyarrhythmia with exercise, as well as pregnancy, due to findings of potential fetal harm in animal studies. Concomitant use of strong CYP2C19 or CYP3A4 inhibitors (like omeprazole) and other specific heart medications (like disopyramide or a beta-blocker/verapamil combination) was also prohibited [45,48].

Preclinical and clinical data suggest that mavacamten promotes favorable reverse remodeling, including reductions in left ventricular wall thickness and left atrial size. However, a critical unanswered question is whether these structural improvements are sustained after treatment discontinuation or if they reverse upon cessation of therapy, similar to the rapid return of hemodynamic and symptomatic benefits. This uncertainty has implications for understanding whether mavacamten induces a true disease-modifying effect or merely suppresses the hypercontractile phenotype reversibly. If remodeling reverses upon drug withdrawal, it could imply that continuous therapy is required to maintain the benefits, which carries pharmacoeconomic and long-term safety implications. Conversely, sustained remodeling after a finite treatment period would suggest a more profound modification of the disease pathway. Long-term studies with planned drug interruption are needed to distinguish between these possibilities and to determine if the observed remodeling is adaptive or could become maladaptive under certain conditions. A consolidated summary of the key safety considerations, adverse events, and clinical management strategies discussed in this section is provided in Table 1.

## 9. Patient-Reported Outcomes

Patient-reported outcomes (PROs) represent a crucial dimension of mavacamten’s therapeutic benefit, providing direct insight into how treatment affects patients’ daily lives, functional capacity, and overall well-being beyond objective clinical measurements [67]. KCCQ has served as the primary PRO instrument across major clinical trials, providing standardized assessment of symptom frequency, symptom burden, physical limitations, social limitations, and overall quality of life in HCM patients [67].

The EXPLORER-HCM trial demonstrated substantial improvements in patient-reported health status with mavacamten treatment, as measured by clinically meaningful increases in KCCQ-CSS [68]. Patients receiving mavacamten showed significantly greater improvements in KCCQ-CSS compared to placebo, with mean improvements exceeding the minimal clinically important difference of 5 points established for this instrument [52]. These improvements reflected meaningful enhancements across multiple domains of health status, including physical limitations, symptom frequency, quality of life, and social functioning that directly impact patients’ ability to participate in normal daily activities.

Subgroup analyses of patient-reported outcomes have revealed important insights into differential treatment responses across various patient populations and clinical characteristics [50]. Women enrolled in EXPLORER-HCM demonstrated greater improvements in KCCQ-CSS scores compared to men, with mean improvements of 14.8 points versus 6.1 points respectively, suggesting potential gender-related differences in PRO responses to mavacamten therapy [50]. These differential responses may reflect baseline differences in symptom severity, disease manifestation, or treatment responsiveness between men and women with HCM, highlighting the importance of individualized treatment approaches and outcome assessment.

The relationship between patient-reported outcomes and objective clinical measurements has been systematically evaluated to understand how subjective treatment benefits correlate with measurable physiological improvements [52]. Analyses from EXPLORER-HCM have demonstrated that changes in echocardiographic parameters, including LVOT gradient reduction and improvements in diastolic function measures, correlate with improvements in KCCQ scores, suggesting that objective hemodynamic improvements translate into meaningful patient-perceived benefits [52]. However, the correlation is not perfect, indicating that PROs capture important aspects of treatment benefit that may not be fully reflected in traditional clinical measurements.

Long-term patient-reported outcome data from extension studies have demonstrated sustained improvements in health status and quality of life measures with continued mavacamten therapy [41]. The PIONEER-OLE study showed continued improvements in KCCQ Overall Summary Scores over more than 200 weeks of treatment, with mean improvements of 17 points from baseline representing substantial and sustained enhancement in patient-reported health status [41]. These long-term PRO improvements suggest that mavacamten’s benefits are not merely transient symptomatic relief but represent meaningful and durable enhancements in patients’ functional capacity and quality of life.

Disease-specific PRO instruments beyond the KCCQ have been employed to capture HCM-specific aspects of symptom burden and functional limitation [69]. The Hypertrophic Cardiomyopathy Symptom Questionnaire (HCMSQ), particularly its Shortness of Breath subscore, has provided additional insights into respiratory symptom improvements with mavacamten treatment [68]. These specialized instruments complement broader quality of life measures by focusing on symptoms and limitations that are particularly relevant to HCM patients, providing more sensitive detection of treatment benefits in this specific patient population.

The clinical significance of PRO improvements has been validated through anchor-based analyses that relate changes in patient-reported measures to clinically meaningful outcomes [52]. Studies have established that improvements in KCCQ scores of 5 points or greater represent minimal clinically important differences, while improvements of 10–15 points or more represent moderate to large clinical benefits [67]. The magnitude of PRO improvements observed with mavacamten treatment consistently exceeds these thresholds, confirming that the statistical significance of treatment effects translates into meaningful clinical benefits for patients.

Cross-cultural validation of patient-reported outcomes has been important for establishing mavacamten’s benefits across diverse patient populations and healthcare systems [49]. The EXPLORER-CN trial in Chinese patients demonstrated PRO improvements consistent with those observed in global populations, despite potential cultural and linguistic differences in symptom expression and health status perception [49]. These findings support the universal applicability of mavacamten’s patient-reported benefits across different ethnic and cultural contexts.

The economic implications of patient-reported outcome improvements have been evaluated through utility assessments and quality-adjusted life year calculations [70]. The EQ-5D-5L quality of life instrument has been employed in clinical trials to capture health state utilities that can be incorporated into health economic evaluations [70]. Mavacamten treatment has been associated with improvements in EQ-5D-5L index scores and Visual Analogue Scale ratings that support the drug’s cost-effectiveness by demonstrating meaningful quality of life enhancements that contribute to overall health utility.

The integration of patient-reported outcomes into clinical decision-making represents an important advancement in personalized HCM care enabled by mavacamten therapy [67]. Clinicians can now use validated PRO instruments to assess treatment response, guide dose adjustments, and optimize therapeutic outcomes based on patient-reported symptoms and functional status [34]. This patient-centered approach to treatment monitoring complements traditional clinical assessments and may improve treatment satisfaction and adherence by incorporating patients’ perspectives into therapeutic decision-making.

Patients experiencing significant improvements in KCCQ scores may have reduced needs for emergency department visits, hospitalizations, and other healthcare services, creating potential cost offsets that contribute to mavacamten’s overall economic value proposition. These relationships between PROs and healthcare utilization provide important data for health economic modeling and value-based care initiatives.

## 10. Conclusions

Mavacamten represents a transformative advancement in the management of obstructive hypertrophic cardiomyopathy, introducing a novel, mechanism-based therapeutic modality that directly attenuates sarcomeric hypercontractility, the central pathophysiologic driver of disease. Through selective, reversible inhibition of β-cardiac myosin ATPase, mavacamten not only ameliorates symptomatic burden and functional limitations but also promotes favorable cardiac remodeling and improves diastolic function, effects supported by robust clinical trial data. The integration of pharmacogenomic insights, particularly CYP2C19 genotype-dependent metabolism, facilitates refined patient stratification and dosing precision. While mavacamten’s approval marks a seminal milestone, ongoing longitudinal studies and diverse population assessments are crucial to elucidate its long-term impact on mortality, heart failure progression, and arrhythmogenic risk. Moreover, mavacamten’s success predicates a promising era for sarcomere-targeted therapies, expanding the horizon of precision cardiovascular medicine. Ultimately, mavacamten exemplifies how deep molecular understanding of cardiac pathobiology can be harnessed to develop disease-specific, efficacious, and safe therapies that redefine treatment standards for complex inherited cardiomyopathies. Key mechanistic gaps include (1) the impact of long-term myosin inhibition on myocardial energetics (PCr/ATP), (2) effects on sarcomere protein turnover and hypertrophic signaling pathways, (3) interaction with microvascular/ischaemic substrates, and (4) molecular determinants of variable response across genotypes—each of them is an attractive target for translational studies comprising metabolic imaging, proteomics, and biopsy-based analyses. All of the above mentioned will be crucial for optimizing therapy and developing next-generation sarcomere targeting agents.

## Figures and Tables

**Table 1:** Summary of Mavacamten’s Key Safety Considerations and Adverse Events.

Safety Category	Specific Issue	Frequency/Incidence	Clinical Management
REMS Requirement	Monitoring for LVEF	Mandatory echocardiograms at 4, 8, and 12 weeks, then every 12 weeks	Ensures early detection of systolic dysfunction
Cardiac	Reduction in LVEF (<50%)	~10% of patients (typically transient)	Dose reduction or temporary interruption; usually reversible
	Heart Failure	1.42 per 100 patient-years (aligned with natural history)	Standard heart failure management, dose adjustment
	Atrial Fibrillation (new onset)	1.89 per 100 patient-years (aligned with natural history)	Standard rhythm management; not clearly drug-induced
Gastrointestinal	Nausea, Diarrhea, Abdominal Discomfort	Common (>1–10%), generally mild	Typically, self-limiting or manageable with supportive care
Neurological	Dizziness, Headache	Common (>1–10%), often transient	May be related to hemodynamic changes; usually does not require discontinuation
Other	Fatigue	Common	Dose adjustment may be considered if severe
Treatment Discontinuation	Due to Adverse Events	3.5% of the population treated in clinical trials	Permanently discontinue if LVEF remains low or heart failure symptoms persist despite adjustment

## Supplementary Materials

The following supporting information can be downloaded at: https://www.mdpi.com/article/10.3390/jcm14238594/s1, Table S1: Key Clinical Trials Evaluating Mavacamten in Hypertrophic Cardiomyopathy; Table S2: Pharmacokinetics and Pharmacogenomics of mavacamten. References [16,18,27,40,41,43,45,46,47,48,54,71] are cited in the supplementary materials.
