# Supplementary material for: Mavacamten for Obstructive Hypertrophic Cardiomyopathy: Targeting Sarcomeric Hypercontractility with Demonstrated Long-Term Safety and Efficacy—A Narrative Review
Source: J Clin Med. 2025 Dec 4;14(23):8594. doi: 10.3390/jcm14238594 (PMC12693540; doi:10.3390/jcm14238594)
Supplement: Supplementary file 1 [file jcm-14-08594-s001.zip › jcm-3972604-supplementary.pdf]

Supplementary tables:

Table S1. Key Clinical Trials Evaluating Mavacamten in Hypertrophic Cardiomyopathy

Trial Name	Patient Population	Study Design	Primary Endpoint	Key Secondary Outcomes	Results Summary	Reference
ODYSSEY-HCM	580 nonobstructive HCM Patient	international phase 3, double-blind and placebo-controlled	peak oxygen consumption from the start of the study to week 48, change in KCCQ-CSS	change from baseline in minute ventilation/carbon dioxide production slope to week 48, change from baseline in NT-proBNP to week 48.	no greater ancement in exercise capacity, no greater reduction in symptoms according to the KCCQ-CSS, compared to a placebo. 42 patients (14.6%) in mavacamten group did not respond well to mavacamten.	[40]
PIONEER-HCM	21 patients with obstructive HCM patients	open-label proof-of-concept Phase 2 trial	change in postexercise LVOT gradient at 12 weeks	changes in peak oxygen consumption (pVO_2), resting and Valsalva LVOT gradients, left ventricular ejection fraction (LVEF), and numerical rating scale dyspnea score.	demonstrated significant improvements in left ventricular outflow tract gradients, exercise capacity, and patient-reported outcomes, no serious Adeverse effects related to mavacamten were reported, with 1 patient discontuing the study.	[71]

**PIONEER-OLE**	13 patients who completed the PIONEER-HCM trial.	Open-label extension study.	assess the long-term safety and tolerability of mavacamten	assess the long-term effectiveness of mavacamten on obstructive HCM symptoms, LVOT gradients, cardiovascular biomarkers, and echocardiographic measures of structure and function.	demonstrated sustained therapeutic benefits with continued improvements in KCCQ and maintenance of reduced LVOT gradients over years of treatment. 1 patient (7.69) discontinued due to serious adverse event unrelated to drug	[41]

EXPLORER-HCM	251 Adults with symptomatic NYHA II-III oHCM	Randomized, double-blind, placebo-controlled	Change in peak oxygen consumption (pVO_2) and NYHA functional class	LVOT gradient reduction, KCCQ score, NT-proBNP, troponin I	Significant improvement in pVO_2, >50% patients improved NYHA class; substantial LVOT gradient reduction and biomarker improvements. tolerability was comparable to placebo. 1 Patient died unexpectedly in the placebo group. 4 patients (%1.6) discontinued due to adverse effects.	[27]
MAVA-LTE (Long-Term Extension)	following 231 patients from both EXPLORER-HCM and MAVERICK-HCM	Long-Term Extension	Long-term safety and efficacy.	-	sustained therapeutic benefits with continued improvements in symptoms, functional capacity, and hemodynamic parameters over nearly three and a half years of treatment. 3 patients (1.3%) required permanent discontinuation	[45]

**VALOR-HCM**	112 patients who were deemed eligible for septal reduction therapy	Crossover study	patients who proceeded to SRT, eligible for SRT, and whose SRT status could not be evaluated;	changes from baseline in postexercise LVOT gradient, (KCCQ-23 CSS), (NT-proBNP) level, and cardiac troponin I level	remarkable ability to obviate the need for septal reduction therapy, 3 patients (2.8%) permanently discontinued due to adverse events.	[18,46]
MAVERICK-HCM	59 Adults with non-obstructive HCM	Phase 2, randomized, placebo-controlled	improvements in the primary endpoint of peak oxygen consumption	Biomarkers (troponin I), peak VO_2.	No significant peak VO_2 change; significant biomarker reductions suggesting potential benefit, medication was well tolerated (no differences in reported serious adverse events between treatment groups).	[43, 47, 71]

**EXPLORER-CN**	81 Chinese patients with symptomatic oHCM	randomized, double-blind, placebo-controlled study	Change from baseline to week 30 in Valsalva (LVOT)	change in resting LVOT peak gradient, proportion of participants achieving a Valsalva LVOT peak gradient <30 or < 50 mm Hg, NYHA functional class improvement, change in KCCQ- CSS, and cardiac biomarkers and left ventricular mass index.	Efficacy and safety consistent with the global EXPLORER-HCM population, with significant improvements in LVOT gradients and echocardiographic parameters. 2 Patients (2.46%) stopped therapy early.	[48]

**SCOUT-HCM**	40 adolescent patients with HCM	Phase 3 randomized, double-blind, placebo-controlled study	changes in Valsalva LVOT gradient	echocardiographic parameters, safety assessments, pharmacokinetic characterization, and exercise capacity measurements	-	[54]

Long-term Extension Studies	Varied HCM populations	Open-label extension	Safety, tolerability, long-term efficacy	Echocardiographic remodeling, symptom relief	Durable efficacy with sustained LV wall thickness reduction and improved diastolic function; generally favorable safety profile	[16]

Table S2. Pharmacokinetics and Pharmacogenomics of mavacamten

Parameter	Description	Clinical Implication
Absorption	Oral, immediate release capsules; optimized for bioavailability	Reliable plasma concentrations enabling flexible dosing
Metabolism	Primarily hepatic via CYP2C19; minor contribution by CYP3A4	CYP2C19 phenotype significantly affects drug clearance
Clearance (CL/F)	Variable: Poor metabolizers (PM) have markedly reduced clearance	PMs require lower doses to avoid toxicity
Elimination Half-life	~6–15 hours (normal metabolizers), 23–37 hours (poor metabolizers)	Adjust dose interval based on CYP2C19 genotype

Pharmacogenomics	CYP2C19 poor, intermediate, normal, ultra-rapid metabolizers	Personalized dosing critical for safety and efficacy
Special populations	Minimal effect of age, gender, renal impairment; significant hepatic impairment affects clearance	Hepatic impairment dosing adjustments necessary
Drug-Drug Interactions	Potential interactions with CYP2C19 and CYP3A4 inhibitors	Requires monitoring and dose adjustment in polypharmacy
